# More than just an intermediate: hydrogen sulfide signalling in plants

**DOI:** 10.1093/jxb/erx352

**Published:** 2017-10-13

**Authors:** Milos R Filipovic, Vladimir M Jovanović

**Affiliations:** 1 University of Bordeaux, IBGC, UMR 5095, Bordeaux, France; 2 CNRS, IBGC, UMR 5095, Bordeaux, France; 3 Institute for Biological Research ‘Siniša Stanković’, University of Belgrade, Belgrade, Serbia

**Keywords:** Hydrogen sulfide, persulfidation, proteomics, redox regulation, sulfenylation, sulfur assimilation

## Abstract

This article comments on:

**Aroca A, Benito JM, Gotor C, Romero LC.** 2017. Persulfidation proteome reveals the regulation of protein function by hydrogen sulfide in diverse biochemical processes in Arabidopsis. Journal of Experimental Botany **68,** 4915–4927.

**Hydrogen sulfide (H_2_S) has come a long way from air pollutant, to intermediate in sulfur assimilation, to signalling molecule. Using a proteomic approach, Aroca *et al.* (2017) investigated post-translational modifications of cysteine residues (protein persulfidation) controlled by H_2_S to show that at least 5% of the entire proteome encoded by the Arabidopsis genome is persulfidated and, completing the journey, that H_2_S is indeed a signalling molecule in plants.**

Hydrogen sulfide (H_2_S) is commonly considered as a pollutant and as such has long been known to plant scientists as well as the general public. However, life emerged in an H_2_S-rich atmosphere, with H_2_S and cyanide being the only chemicals pre-requisite for the formation of all biomolecule precursors in prebiotic Earth (Patel *et al.*, 2015). H_2_S was also the first electron donor (before water replaced it) used for bacterial photosynthesis, remnants of which can be found in modern-day anaerobic photosynthetic purple and green sulfur bacteria. While its production in plants during sulfur assimilation has been known for quite a while (Box 1), our knowledge of the signalling role of H_2_S only started emerging recently (Lisjak *et al.*, 2013; Calderwood *et al.*, 2014). Being produced on demand and involved in regulation of blood pressure and neurotransmission, among other processes, H_2_S acts as a true gasotransmitter in animals (Paul and Snyder, 2015). H_2_S and its donors show promising therapeutic potential (Szabo, 2007) and could put small animals into a suspended animation-like state (Blackstone *et al.*, 2005). Therefore, it could be that signalling by H_2_S is a universal phenomenon conserved among all kingdoms of life.

**Box 1. Hydrogen sulfide in plants**

Hydrogen sulfide is produced by plant cells as an intermediate of assimilatory sulfate reduction (left panel). Plants are able to reduce activated sulfate to sulfite and then sulfite reductase catalyses the further reduction of sulfite to H_2_S. These reactions take place in plastids. H_2_S is then incorporated into O-acetylserine (OAS) to form cysteine, in a reaction catalysed by O-acetylserine thiol lyase (OAS-TL). OAS-TL can, however, catalyse the reverse reaction as well, decomposing cysteine to H_2_S and OAS.

Additionally H_2_S can be produced by the action of D-cysteine desulfhydrase (DCDES) and L-cysteine desulfhydrase (DES1), with concomitant formation of ammonia and pyruvate. Furthermore, H_2_S can be released in a reaction catalysed by cyanoalanine synthase (CAS), where cyanide and cysteine are used as substrates and cyanoalanine co-formed with H_2_S.

As shown in the panel on the right, at low doses H_2_S shows a positive effect on germination, size and fresh weight of several plant species, while at high concentration it exerts phytotoxic effects.

**Figure:**
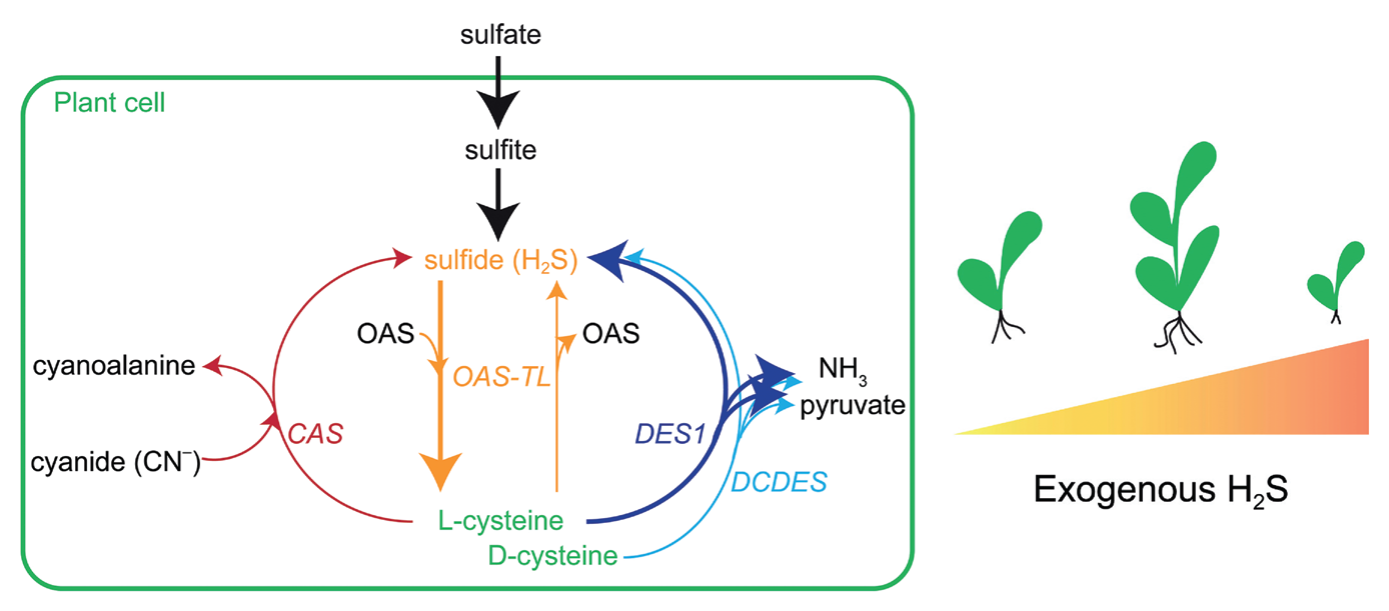

The past decade has witnessed a growing body of evidence confirming a signalling role of H_2_S in plants as well. Although phytotoxic at high concentrations, at low doses H_2_S increases the size and fresh weight of several plant species both in hydroculture and in soil (Dooley *et al.*, 2013). Furthermore, H_2_S showed a positive effect on germination, growth of leaf disks and yield of wheat, suggesting enormous agricultural potential. In addition, H_2_S protects against heavy metal, drought and osmotic stress (e.g. Zhang *et al.*, 2008; Jin *et al.*, 2011). Finally, H_2_S also inhibits autophagy in plants (Alvarez *et al.*, 2012).

Although the biochemistry of H_2_S is quite rich, it has been suggested that oxidative post-translational modification of cysteine residues (RSH) to persulfides (RSSH) is the main way by which H_2_S exerts its numerous biological functions (Filipovic, 2015). In their study, Aroca *et al.* (2017) reveal that at least 5% of the entire proteome encoded by the Arabidopsis genome is persulfidated, and that H_2_S is not just an intermediate product of sulfur assimilation pathways but instead involved in regulation of the redox status of cysteine residues of proteins involved in many classical biochemical pathways.

## Secrets of the plant persulfidome

The reactivity of persulfides is very similar to that of thiols, with persulfides being even better nucleophiles (Cuevasanta *et al.*, 2015), which represents a significant obstacle for designing labelling methods. Relying on this similarity in reactivity some researchers have developed a selective tag-switch method where both thiols and persulfides get initially blocked with the aromatic thiol-blocking reagent, MSBT. The activated disulfide, formed in the reaction of MSBT with persulfide, reacts with a tag-bearing nucleophile, a cyanoacetate derivative (Zhang *et al.*, 2014). Using this methodological approach to detect persulfides in plants, Aroca *et al.* went even further and mined the whole Arabidopsis persulfidome. Besides being the first full-scale proteomic analysis of this oxidative post-translational modification in any species, this study reveals at least 2015 different protein targets as endogenously persulfidated, with 4000 targets found in samples extracted from mature leaves. This observation matches well with the study by Dooley *et al.* (2013), who reported that the bigger the plant, the better it copes with high doses of H_2_S, suggesting that this may be an adaptative response.

Just a first glance at the persulfidome of Arabidopsis points towards important biochemical pathways such as the glycolytic cycle, TCA cycle and starch biosynthesis pathway, as well as Calvin–Benson cycle, suggesting that essential metabolic axes for plant growth and development might be affected by this modification. The actual effect that persulfidation might have on enzyme activity remains to be examined, and one way to address this in future would be the metabolomic approach where changes in metabolites controlled by different enzymes within each cycle or pathway could be monitored. Keeping in mind the agricultural potential that H_2_S might have on increasing crop biomass, the database that Aroca *et al.* (2017) produced represents a valuable starting point for deciphering the actual targets in achieving that goal.

Previous work of Romero and Gotor’s group revealed the role of H_2_S in autophagy, suggesting that H_2_S somehow inhibits this process, preventing Atg8 (autophagy-related ubiquitin-like protein) accumulation (Alvarez *et al.*, 2012). In their current work they found that several partners involved in Atg8 processing (Atg3, Atg5 and Atg7) are endogenously persulfidated (Aroca *et al.*, 2017). Atg7 is an E1-like enzyme and forms a thioester bond with Atg8, hydrolysing ATP. However, persulfidation of the Atg7 active site cysteine would result in an acyl-disulfide link with Atg8, instead of Gly–Cys thioester bond. The fate of that species would probably affect subsequent transfer to Atg3, even more when the latter is also persulfidated, thus inhibiting classical autophagy-inducing stimulus propagation. Furthermore, Aroca *et al.* (2017) found that Atg18a is a target of persulfidation. This enzyme is particularly sensitive to oxidative, salt and osmotic/drought stresses, and important for the regulation of plant defence responses (Xiong *et al.*, 2005), all of which were previously shown to be prevented by H_2_S. The similarity of Atg enzymes with other ligases involved in ubiquitination and sumoylation processes raises the possibility that these processes are also controlled by persulfidation throughout all kingdoms of life.

Finally, Aroca *et al.* (2017) also attempted to understand the role of L-cysteine desulfhydrase 1 (DES1)-catalysed H_2_S production (Box 1) on global protein persulfidation. Only a small number of proteins (47) were found to be down-persulfidated in *des1* mutants when compared to the control; however, among them are very specific targets such as protein kinases and phosphatases, as well as abscisic acid (ABA) receptors PYR1 and PYL1, potentially explaining previous observations that exogenous H_2_S application restores stomatal closure by ABA in *des1* mutants. As H_2_S production is controlled by several enzymes (Box 1), the extent to which activity of these other enzymes affects the overall persulfidome in plant cells remains to be answered.

## Persulfidation in plants: what for?

One big question imposes itself: could it be that the functions of 2015 proteins are regulated by persulfidation? The answer is probably no, and although the stochastic nature of events could be an explanation (due to relatively high H_2_S flux through sulfite reduction), it is more plausible that protein persulfidation is a conserved redox modification used to resolve cysteine oxidation (Box 2). It is important to note that H_2_S cannot modify cysteine residues directly and that some oxidation step is required (Cuevasanta *et al.*, 2015). During the oxidative stress, cysteine residues get oxidized to sulfenic acids (RSOH) which can, if the stress persists, be oxidized further to sulfinic (RSO_2_H) and sulfonic acids (RSO_3_H); these modifications are irreversible (Box 2). Furthermore, if buried deep into protein pockets, sulfenic acids could not easily be reached and reduced back to thiols. Reaction of sulfenic acid with H_2_S is 600 times faster than with glutathione (Ceuvasanta *et al.*, 2015) and increased persulfidation has been observed as a response to H_2_O_2_ stress (Wedmann *et al.*, 2016). It is tempting to speculate that persulfidation is an evolutionary remnant of the times when life emerged in a sulfide-rich environment and that it represents the simplest way to protect proteins from oxidative damage. Even if the stress persisted, persulfides would be good scavangers of reactive oxygen species, forming RSSO_3_H, which can easily be reduced back to restore free thiols (Filipovic, 2015; Wedmann *et al.*, 2016). Indeed, such a hypothesis fits well with observations that H_2_S treatment protects plants from different stressors such as heavy metals (Zhang *et al.*, 2008). Aroca *et al.* (2017) found a large number of persulfidated proteins to be located in chloroplasts, where most H_2_S production takes place during the sulfur assimilation process and where substantial production of reactive oxygen species also occurs (Inupakutika *et al.*, 2016). Persulfides could easily be reduced back to thiols by the action of thioredoxin, restoring the bound H_2_S (Wedmann *et al.*, 2016). In this way, H_2_S gets recycled and reused by the cell.

**Box 2. Protein persulfidation and its postulated role in protection**

Protein persulfidation (PSSH) is a post-translational modification of cysteine residues (PSH), much like S-nitrosation (PSNO, caused by NO) and sulfenylation (PSOH, caused by H_2_O_2_). Furthermore, all these modifications seem to be interconvertable with different outcomes on protein structure and function (left panel).

Protein persulfidation can serve as a mechanism, on the one hand, to reduce back oxidized cysteine residues and, on the other, to increase the antioxidant capacity of thiol pools (right panel). Hence, upon exposure to reactive oxygen species, thiol oxidation that initially starts with formation of sulfenic acids (RSOH, still reversible modification) could proceed further with formation of irreversible sulfinic (RSO_2_H) and sulfonic acids (RSO_3_H); H_2_S could react with sulfenic acid to form persulfides and prevent this oxidation; and persulfides could be reduced back to thiols by the action of the thioredoxin sytem. The p*K*_a_ of persulfides is lower than that of corresponding thiols, suggesting that at physiological conditions, the majority of persulfide would be in deprotonated form (RSS^–^), making the persulfide ‘super’ nucleophilic. So, if the exposure to reactive oxygen species proceeds, persulfidated protein will react faster with ROS/RNS and form an adduct (RSSO_3_H) that could also be cleaved by thioredoxin to restore free thiol.

**Figure:**
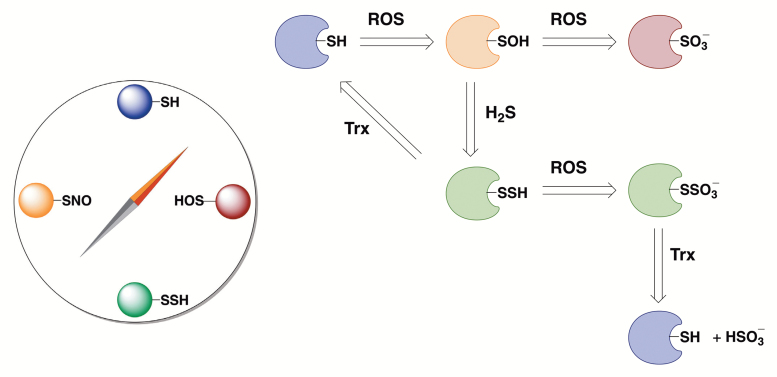

## Stepping forward

Although the path has been paved by establishing the database of persulfidated proteins, many questions, ranging from very fundamental to applied, remain to be answered.

How does H_2_S cause protein persulfidation? Are there enzymes that could serve as ‘persulfidases’ and therefore show target specificity? What is the role of metalloproteins and reactive sulfur species produced following reaction with H_2_S? Which enzymes other than thioredoxin could act as de-persulfidases? These are fundamental questions that, depending on the answers, could place persulfidation as a level of post-translational regulation equivalent to phosphorylation.

It is also of utmost importance to understand the cross-talk between nitric oxide (NO) and H_2_S, both gasotransmitters. This has been studied (bio)chemically, but always in the context of animal physiology. The strong correlation between S-nitrosation, post-translational modification controlled by NO and persulfidation observed by Aroca *et al.* (2017) warrants further examination. Are these modifications mutually exchangeable? What is the spatio-temporal distribution of these modifications in plant cells and what are the biological outcomes on the same targets? The same questions could be applied to other cysteine modifications such as sulfenylation, glutathionylation or S-acylation.

The functional role that persulfidation might have on specific targets needs further validation as well. Aroca *et al.* (2017) showed persulfidation of major enzymes involved not just in carbon assimilation, but in nitrogen assimilation as well. Knowledge of how H_2_S controls all these pathways could be used to achieve increases in photosynthesis and consequently biomass yields in the agricultural field. Furthermore, deciphering the role of persulfidation on production of secondary metabolites in specific species could prove valuable in controlling yields.

Similarly to humans, where the restoration of disease-caused decreases in persulfidation is shown to be curative, it is possible that persulfidation in plants could be involved in protection against pathogens such as fungi. This process is normally controlled by jasmonate, and almost half of the jasmonate biosynthesis-related proteins were found to be persulfidated by Aroca *et al*. Understanding how persulfidation changes under pathogen-induced stress would result in identification of useful targets that could be manipulated to increase plant resistance to different stressors. Furthermore, as jasmonate is involved in regulation of protein storage or tuber formation in plants, persulfidation induced by exogenous sulfide could influence those processes and may prove useful in agricultural practice. So there is no doubt that this study is just a stepping stone towards more exciting research to come.
